# Inadequate Dissemination of Phase I Trials: A Retrospective Cohort Study

**DOI:** 10.1371/journal.pmed.1000034

**Published:** 2009-02-17

**Authors:** Evelyne Decullier, An-Wen Chan, François Chapuis

**Affiliations:** 1 Hospices Civils de Lyon, Pôle Information Médicale et Evaluation en Recherche, Lyon, France; 2 Université de Lyon, Laboratoire Santé Individu Société (EA SIS), Lyon, France; 3 Université Lyon 1, Lyon, France; 4 Comité de Protection des Personnes, CPP Sud-Est III, Lyon, France; 5 Mayo Clinic, Rochester, Minnesota, United States; 6 Université Lyon 1, Réseau d'Épidémiologie Clinique International Francophone (RECIF), Lyon, France; Nordic Cochrane Centre, Denmark

## Abstract

**Background:**

Drug development is ideally a logical sequence in which information from small early studies (Phase I) is subsequently used to inform and plan larger, more definitive studies (Phases II–IV). Phase I trials are unique because they generally provide the first evaluation of new drugs in humans. The conduct and dissemination of Phase I trials have not previously been empirically evaluated. Our objective was to describe the initiation, completion, and publication of Phase I trials in comparison with Phase II–IV trials.

**Methods and Findings:**

We reviewed a cohort of all protocols approved by a sample of ethics committees in France from January 1, 1994 to December 31, 1994. The comparison of 140 Phase I trials with 304 Phase II–IV trials, showed that Phase I studies were more likely to be initiated (133/140 [95%] versus 269/304 [88%]), more likely to be completed (127/133 [95%] versus 218/269 [81%]), and more likely to produce confirmatory results (71/83 [86%] versus 125/175 [71%]) than Phase II–IV trials. Publication was less frequent for Phase I studies (21/127 [17%] versus 93/218 [43%]), even if only accounting for studies providing confirmatory results (18/71 [25%] versus 79/125 [63%]).

**Conclusions:**

The initiation, completion, and publications of Phase I trials are different from those of other studies. Moreover, the results of these trials should be published in order to ensure the integrity of the overall body of scientific knowledge, and ultimately the safety of future trial participants and patients.

## Abstract 2

François Chapuis and colleagues examine a cohort of clinical trial protocols approved by French ethics committees, and show that Phase I trials are less frequently published than other types of trials.

## Editors' Summary

**Background.:**

Before a new drug is used to treat patients, its benefits and harms have to be carefully investigated in clinical trials—studies that investigate the drug's effects on people. Because giving any new drug to people is potentially dangerous, drugs are first tested in a short “Phase I” trial in which a few people (usually healthy volunteers) are given doses of the drug likely to have a therapeutic effect. A Phase I trial evaluates the safety and tolerability of the drug and investigates how the human body handles the drug. It may also provide some information about the drug's efficacy that can guide the design of later trials. The next stage of clinical drug development is a Phase II trial in which the therapeutic efficacy of the drug is investigated by giving more patients and volunteers different doses of the drug. Finally, several large Phase III trials are undertaken to confirm the evidence collected in the Phase II trial about the drug's efficacy and safety. If the Phase III trials are successful, the drug will receive official marketing approval. In some cases, this approval requires Phase IV (postapproval) trials to be done to optimize the drug's use in clinical practice.

**Why Was This Study Done?:**

In an ideal world, the results of all clinical trials on new drugs would be published in medical journals so that doctors and patients could make fully informed decisions about the treatments available to them. Unfortunately, this is not an ideal world and, for example, it is well known that the results of Phase III trials in which a new drug outperforms a standard treatment are more likely to be published than those in which the new drug performs badly or has unwanted side effects (an example of “publication bias”). But what about the results of Phase I trials? These need to be widely disseminated so that researchers can avoid unknowingly exposing people to potentially dangerous new drugs after similar drugs have caused adverse side effects. However, drug companies are often reluctant to disclose information on early phase trials. In this study, the researchers ask whether the dissemination of the results of Phase I trials is adequate.

**What Did the Researchers Do and Find?:**

The researchers identified 667 drug trial protocols approved in 1994 by 25 French research ethics committees (independent panels of experts that ensure that the rights, safety, and well-being of trial participants are protected). In 2001, questionnaires were mailed to each trial's principal investigator asking whether the trial had been started and completed and whether its results had been published in a medical journal or otherwise disseminated (for example, by presentation at a scientific meeting). 140 questionnaires for Phase I trials and 304 for Phase II–IV trials were returned and analyzed by the investigators. They found that Phase I trials were more likely to have been started and to have been completed than Phase II–IV trials. The results of 86% of the Phase I studies matched the researchers' expectations, but the study hypothesis was confirmed in only 71% of the Phase II–IV trials. Finally, the results of 17% of the Phase I studies were published in scientific journals compared to 43% of the Phase II–IV studies. About half of the Phase I study results were not disseminated in any form.

**What Do These Findings Mean?:**

These findings suggest that the fate of Phase I trials is different from that of other clinical trials and that there is inadequate dissemination of the results of these early trials. These findings may not be generalizable to other countries and may be affected by the poor questionnaire response rate. Nevertheless, they suggest that steps need to be taken to ensure that the results of Phase I studies are more widely disseminated. Recent calls by the World Health Organization and other bodies for mandatory preregistration in trial registries of all Phase I trials as well as all Phase II–IV trials should improve the situation by providing basic information about Phase I trials whose results are not published in full elsewhere.

**Additional Information.:**

Please access these Web sites via the online version of this summary at http://dx.doi.org/10.1371/journal.pmed.1000034.

- Two recent research articles published in *PLoS Medicine*—by Ida Sim and colleagues (*PLoS Med* e191) and by Lisa Bero and colleagues (*PLoS Med* e217)—investigate publication bias in Phase III trials
- The ClinicalTrials.gov Web site provides information about the US National Institutes of Health clinical trial registry, background information about clinical trials, and a fact sheet detailing the requirements of the US Food and Drug Administration (the body that approves drugs in the USA) Amendments Act 2007 for trial registration
- The World Health Organization's International Clinical Trials Registry Platform is working toward setting international norms and standards for the reporting of clinical trials (in several languages)

## Introduction

New drugs are developed following a logical, step-by-step procedure in which findings from small early studies are used to inform and design subsequent larger studies [1]. To develop new drugs efficiently, it is essential to identify their properties in the early stages of development, and then construct a research plan adapted to these characteristics [1,2].

The different stages of clinical trials involving pharmaceutical interventions are classified into four phases (Box 1) [1,2]. Phase I trials provide an early evaluation of short-term safety, tolerability, pharmacokinetics, and sometimes efficacy. Phase II studies explore therapeutic efficacy in patients, whilst Phase III studies aim to confirm therapeutic benefit. Phase IV trials are designed to optimize the drug's use in clinical practice. These definitions involve a degree of subjective judgment, and substantial overlap exists between terms. More recently, a fifth phase was created for exploratory first-in humans studies without therapeutic or diagnostic intent [3].

In France, all trials involving human beings must be submitted to a research ethics committee (REC) by the principal investigator in accordance with the Huriet-Sérusclat Act of 1988 [4]. Phase I trials are more closely monitored than other phases of drug development because they constitute the first introduction of a new drug undergoing investigation in humans. Moreover, they are usually conducted in healthy volunteers to assess their safety [4], although new therapies for some diseases such as cancer or HIV infection are understandably often first investigated in patients.

A large number of reviews have previously assessed the fate of research studies and publication bias but to our knowledge, none have been focused on Phase I trials across specialized fields [5–8]. We reviewed a cohort of clinical trial protocols in France to describe the initiation, completion, and publication of Phase I trials, and to determine whether these outcomes differed from those of Phase II–IV trials.

## Methods

### Study Cohort

In 1994, there were 48 RECs in France; each REC was responsible for a specific administrative geographical area, was evaluating equivalent types of protocols, and was representative of the biomedical research carried out locally. The principal investigator (FC) selected 25 RECs on the basis of geographic criteria to ensure a representative cross-section. Consent was obtained from the chairperson of all selected committees to participate in the study.

We included all pharmaceutical trials (i.e., studies that prospectively assigned drug interventions to humans to evaluate their effects on health outcomes) that were newly approved between January 1, 1994 and December 31, 1994 by any of the 25 participating French committees. In France, each trial is submitted by the principal investigator to only one REC nationally, it was therefore impossible for a trial to be counted twice in our cohort. No sample size calculation was made.

### Data Collection

Each REC was responsible for collecting its own data from committee files and questionnaires were sent to investigators. A research assistant from each REC attended a formal training session on the abstraction of study characteristics in June 2000. An identification number was assigned to each trial protocol to ensure anonymity, and all collected data were then forwarded to the coordinating centre where the data were checked and analyzed. RECs were contacted for any missing or incorrect data. Anonymity was not preserved in cases where the investigator provided full journal references.

**Box 1**

International Conference on Harmonization of Definitions of Trial Phases for Pharmaceutical Trials [1,2]

**Phase I**

Studies conducted in Phase I typically involve one or a combination of the following objectives:

(a) to determine the tolerability of the dose range expected and the nature of adverse reactions that can be expected;

(b) to characterize a drug's absorption, distribution, metabolism, and excretion and also to assess the clearance of the drug and to anticipate possible accumulation of potential efficacy that may guide the dosage and dose regimen;

(c) to provide early estimates of activity and potential efficacy that may guide the dosage and dose regimen in later studies;

(d) to provide an early measurement of drug activity when drug activity is readily measurable with a short duration of drug exposure in patients at this early stage.

Phase I studies involve a small number of persons.

**Phase II**

The primary objective of Phase II studies is to explore therapeutic efficacy in patients. Phase II studies are typically conducted in a group of patients who are selected by relatively narrow criteria, leading to a relatively homogeneous population, and who are closely monitored.

An important goal for this phase is to determine the dose(s) and regimen for Phase III trials.

Additional objectives of clinical trials conducted in Phase II may include evaluation of potential study endpoints, therapeutic regimens (including concomitant medications), and target populations (e.g., mild versus severe disease) for further study in Phase II or III.

These studies are performed on several hundred volunteers, including a limited number of patients with the target disease or disorder.

**Phase III**

The primary objective of Phase III studies is to demonstrate or confirm therapeutic benefit. Studies in Phase III are designed to confirm the preliminary evidence accumulated in Phase II that a drug is safe and effective for use in the intended indication and recipient population. These studies are intended to provide an adequate basis for marketing approval. Studies in Phase III may also further explore the dose–response relationship, or explore the drug's use in wider populations, in different stages of disease, or in combination with another drug.

These studies are performed on groups of patients large enough to identify clinically significant responses.

**Phase IV**

Phase IV studies after-drug approval and marketing and are often important for optimizing the drug's use. Commonly conducted studies include additional drug interaction, dose–response, or safety studies and studies designed to support use under the approved indication, e.g., mortality/morbidity studies, epidemiological studies.

Data were collected from committee files, including trial protocols, about the type of investigator, sponsor, participant population (healthy volunteers versus patients), study phase, intervention, design, study sites, estimated sample size, duration, and amendments after trial initiation.

**Box 2. Definitions Used**

Direction of results: Investigator had to classify their study as a whole: no hypothesis tested, results not known, results confirming study hypothesis (“confirmatory results”), results invalidating study hypothesis (“invalidating results”), results neither confirming nor invalidating study hypothesis (“inconclusive results”).
Publication: Study published as a full article in a general or specialized peer-reviewed journal.
Grey literature: Studies published in formats other than scientific papers and not generally accessible through libraries (internal reports, theses, abstracts, posters) [9]. Book chapters were included as grey literature.
Dissemination: Any kind of results communication (scientific papers and grey literature).
Publication bias: The tendency to publish research based on the direction and statistical significance of study findings [10].

Using questionnaires mailed to the principal investigators in January 2001, we also collected follow-up data on funding, trial status (initiated or completed), the actual duration of the study and sample size, study results (confirmation or invalidation of the study hypothesis, inconclusive, or no hypothesis tested), format for dissemination (scientific article, oral presentation, internal report, book chapter, doctoral dissertation, or abstract), and reasons for not publishing. Definitions of terms are provided in Box 2 [9,10]. In cases where no response was forthcoming, principal investigators were contacted up to six times by mail or phone. Where no answer could be obtained, the REC contacted the sponsor in August 2002. We recorded the reason for nonresponse. Due to the requirement for the anonymous coding of data, publication status in bibliographic databases was not verified.

### Ethical Considerations

We conducted this study according to the French law on epidemiological and descriptive studies; as there was no intervention on humans, REC approval was not required [4]. We collected data anonymously to respect the confidentiality of investigators and drug companies, and consent was not required as no patient information was collected.

### Statistical Methods

The primary outcome of our study was the proportion of Phase I and Phase II–IV trials published (see definition in Box 2). As secondary outcomes, we evaluated trial characteristics, initiation and completion rates, and time to publication. The magnitude of publication bias was evaluated in an exploratory analysis. A logistic regression for publication was performed to evaluate the interaction between trial phases and results.

We tabulated descriptive data for all variables (medians and 10th–90th percentile range, proportions). We used chi-square or Fisher's exact test to compare categorical data, and Mann-Whitney tests for continuous variables. We excluded studies with missing data from the analysis on the main endpoints.

To compare the time to publication between Phase I and Phase II–IV trials, we performed a Kaplan-Meier survival analysis using the time from the date of approval by the REC to the date of first publication. The date of first publication was the effective date of publication for published studies and date of questionnaire completion for “in-press” studies. Unpublished studies were censored at the questionnaire completion date.

We included all completed studies with a publication date or a censoring date available. To ensure homogeneity in the follow-up, protocols with a time to publication greater than 8 y were censored at 8 y from approval. We used a log-rank test to compare survival curves.

We used SAS software for all analyses, considering associations to be statistically significant when two-sided *p*-values were < 0.05.

## Results

**Figure 1:**
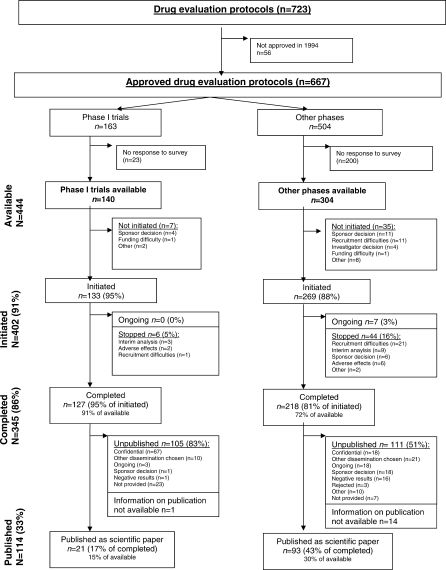
Flow Chart of Studies

In 1994, the 25 ethics committees evaluated 723 drug trial protocols. 56 protocols were excluded for various reasons (approved in 1993 [*n =* 38], withdrawn before approval [*n =* 11], not approved [*n =* 5], or approval not required by law [*n =* 2]). Phase I trials accounted for 24% (*n =* 163) of the remaining 667 protocols (Figure 1).

We received questionnaire responses for 67% (444/667) of the trials, which constituted our final cohort for analysis. Data were unavailable for 223 questionnaires: ten were unsuitable for statistical analysis, 85 investigators did not respond, 49 declined participation, 45 declared that the file was lost, 27 had moved with no identifiable address, and seven had retired. Response rates differed significantly between trial phases: 86% (140/163) for Phase I trials and 60% (304/504) for Phase II–IV trials (*p* < 0.001).

### Trial Characteristics

Administration and conduct differed significantly between Phase I and Phase II–IV trials (Tables 1 and 2). Phase I trials were more frequently led by nonacademic researchers (56% versus 6%), sponsored by industry, conducted in accredited Phase I research units, and conducted at a single study centre. Phase I trials also had a shorter planned duration and smaller estimated sample sizes compared to other trial phases (Table 3). Furthermore, Phase I trials were less likely to use a control group or planned interim analysis.

**Table 1:**
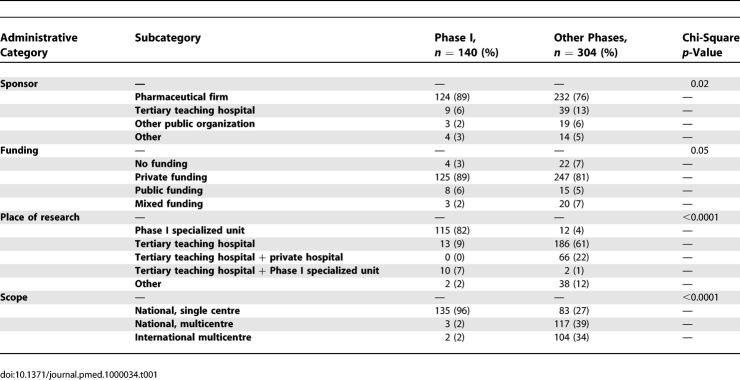
Administration of 444 Clinical Trials

**Table 2:**
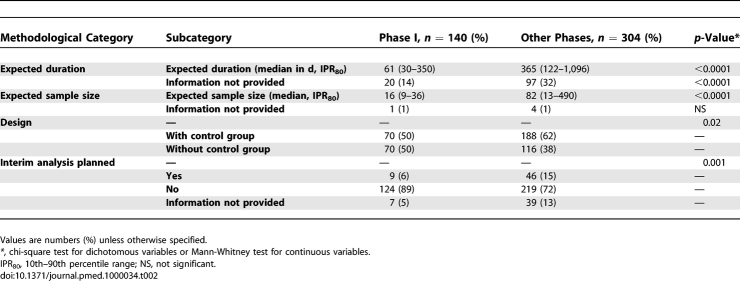
Conduct of 444 Clinical Trials

**Table 3:**
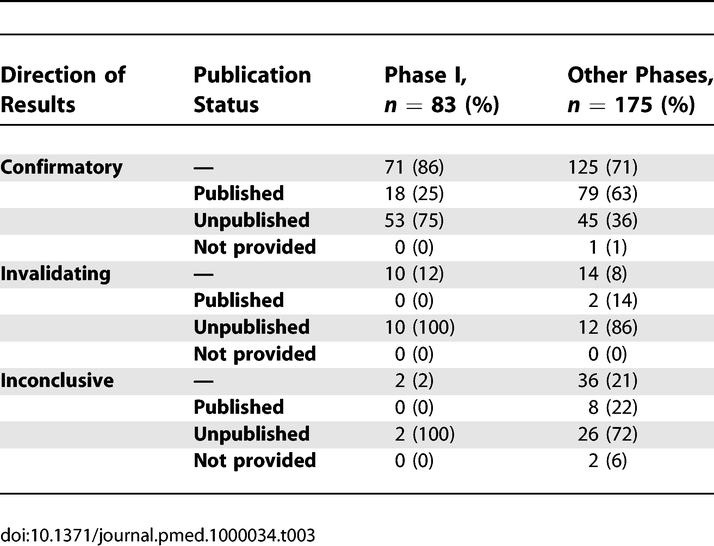
Publication Rates for Completed, Hypothesis-Testing Phase I and Phase II–IV Trials, by Direction of Results

### Initiation and Completion Rates

Approved Phase I trials were more frequently initiated in comparison with other phases (*p* = 0.03) (Figure 1). The main reasons for not beginning a study were mainly the sponsor's decision for Phase I and other phases (Figure 1).

The completion rate for initiated studies was significantly greater (*p* = 0.0004) for Phase I trials compared with other phases (Figure 1). Among the 50 studies that were terminated prematurely, the reasons differed between groups: Phase I studies were mainly stopped because of interim analysis results or adverse effects, whereas other phases were mainly stopped because of recruitment problems (Figure 1).

### Dissemination of the Results of Completed Studies (*n* = 345)

Information on the dissemination of results was provided for 99% (126/127) of the completed Phase I, and 94% (204/218) of completed Phase II–IV trials. Results of Phase I trials were less often published as journal articles than Phase II–IV trials (odds ratio 0.24, 95% confidence interval 0.14–0.41, *p* < 0.0001) (Figure 1).

**Figure 2:**
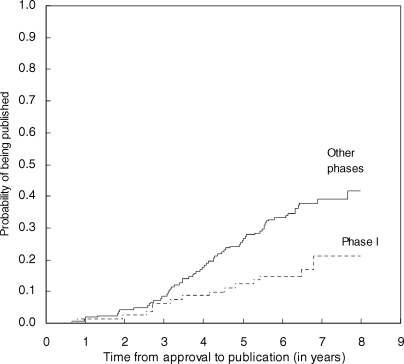
Time from Ethical Approval to Publication for Phase I Versus Phase II–IV Trials

A total of 99 completed studies had to be excluded from the Kaplan-Meier analysis owing to missing information on dates (publication or censoring). Phase I studies took a significantly longer time to be published despite shorter study durations. The medians were not reached but the 10th percentile was, at 4.5 y versus 3.1 y for Phases II–IV (Figure 2 and Table S1).

The reasons provided by principal investigators for nonpublication differed significantly between the two groups (Figure 1): confidentiality was the main reason given for unpublished Phase I studies.

Among the 105 Phase I and 111 Phase II–IV trials that were not published as a full journal article, 33 (31%) and 47 (42%), respectively, were disseminated as grey literature, with internal reports being the most frequent example of this (Table 4).

**Table 4:**
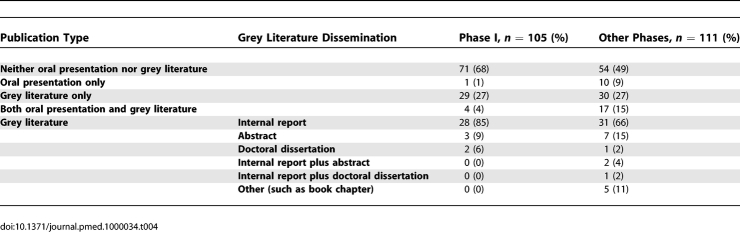
Grey Literature for the 216 Studies Not Published as Scientific Articles

### Nature of Results and Publication Bias

Hypothesis testing was not conducted in 13/127 (10%) of completed Phase I trials and in 10/218 (5%) of completed trials in other phases. Results were unknown or not provided for 31/127 (24%) of Phase I studies compared to 33/218 (15%) of other phases. Information on the direction of results was available for 83/127 (65%) Phase I and 175/218 (80%) Phase II–IV trials. Phase I study results confirmed the investigators' clinical hypotheses more often than other phases (Table 3).

Out of the 258 trials with data available on the direction of results, 255 also had data available on publication status (Table 3). The interaction between phase and results, i.e., differential publication bias, could not be tested as no inconclusive or invalidating Phase I results were published. However the model without interaction was well fitted, which suggests that the interaction would not have been significant.

Trials with confirmatory results had significantly higher publication rates than trials with invalidating or inconclusive results for Phase II–IV trials (64% versus 21%, *p* < 0.0001, exclusion of the three studies with no information on publication), but not significantly for Phase I trials (25% versus 0%, *p* = 0.18).

## Discussion

The main finding of our study was that Phase I studies were more often initiated and completed than other phases, but far less disseminated. Previous studies have described the outcome of study protocols [5–8], but none has focused on Phase I trials across specialties, or compared them with other trial phases. One previous review evaluated the outcome of Phase I trials submitted to an annual oncology meeting and found that 67% were subsequently published [11], but studies submitted to an annual meeting in one specialty field might be not representative of all Phase I studies. Our findings confirm the low overall publication rate of clinical trials found in previous reviews [5–8]. Methodological reviews of trials should therefore consider analyzing different phases as distinct subgroups.

The strength of our study was that we conducted a comprehensive analysis of the pharmaceutical trials approved in 1994 by half of the RECs in France. Approval by local RECs is mandatory for any research protocol involving interventions in human beings. Our sample is therefore representative of the pharmaceutical trials conducted in France.

Several limitations may have affected the results of our study. The overall response rate of 67% is comparable to previous methodological surveys of trials [12,13]. The differential response rate between Phase I and Phase II–IV trials may have introduced response bias, although it is unclear whether this would have influenced our findings in any given direction. It is likely that the publication rates found in our study were overstated if we assume that nonresponders would be more likely to have trials that were not initiated, not completed, or unpublished. On the other hand, we did not confirm the publication status of trials on databases such as PubMed [14] because the files were anonymous. This may have led to an underestimation of publication rates, although our findings were similar to previous reviews [5–8]. Moreover, we were unable to determine the publication date when the specific journal reference was not provided.

Some studies were excluded from the survival analysis due to missing data on dates of publication and dates of censoring, this analysis should therefore be interpreted with caution. We chose to exclude these published studies as the alternative, i.e., including them as censored at time 0, would mean ignoring the fact that they were published. The same also applies to the unpublished studies.

Another practical challenge in describing the methodological characteristics of our cohort was that detailed information was often missing from the protocols, such as the expected study duration. One of the reasons could be related to varying standards in the requirements for protocol content. We focused on the expected duration and sample size as information on the actual duration and sample size was less often available.

The methods and results of our cohort of protocols approved in 1994 are still relevant, since a median delay of 5 to 8 y is generally observed before publication [6]. Moreover, the publication and prospective registration of Phase I trials is still subject to discussion in 2008. The poor dissemination of Phase I trials highlights the importance of their inclusion in the prospective registration of clinical trials [15,16]. Whilst several bodies, including the World Health Organization, the International Committee of Medical Journal Editors, and the Ottawa Group, have called for their registration in public registries [17–20], pharmaceutical companies have expressed their reluctance to disclose information on early phase trials [21].

Although the results of preliminary Phase I studies might be considered by some to be less clinically relevant, there are several ethical and scientific reasons for making this data publicly available. Firstly, if access to medical information is a moral right, then it should be provided irrespective of study funding and phase [22]. The testing of new pharmaceuticals on humans is approved by ethics committees based on the assumption that the inherent risks of trial participation are balanced by the benefit of new scientific knowledge for society. If this knowledge from Phase I remains hidden, then any potential risk incurred by trial participation is excessive and could endanger human lives [23]. Although Phase I trials are usually safe [24] with very few deaths and severe adverse events (incidence of related severe adverse events of 0.2% over a 2-y period among 15,386 participants), adverse events from Phase I studies are known to be rarely published [25], which means that this knowledge is unavailable to other researchers who may unknowingly conduct harmful trials on similar interventions.

The danger of suppressed knowledge was particularly evident in the Phase I trial for TGN1412 [26,27], where the serious adverse reactions suffered by six healthy volunteers in 2006 might have been avoided had the results of a previous unpublished trial of a similar antibody been known [28]. Since this event, reports have been published to highlight the need for improved sharing of knowledge from Phase I trials [29,30]. Publicly accessible trial registers will facilitate the sharing of information on all trials [15,31], including Phase I studies [32]. Furthermore, the willingness of journals to publish Phase I trial results, whether positive or negative, would help improve their unbiased dissemination [33].

It should also be noted that a third of research programs for investigational drugs are abandoned for primarily economic reasons rather than concerns over efficacy or safety [34,35]. This means that even new drugs that show clinical promise are often discontinued and the knowledge lost. Concerns over commercial or competitive advantage should not obstruct transparency and do not outweigh the ethical and scientific responsibilities to disseminate knowledge gained by testing new therapies in humans [26].

To ensure the present and future safety of trial participants and patients as well as the integrity of scientific knowledge, all clinical trials—including Phase I—should be publicly registered at inception and their results disseminated in a timely manner [32].

## Supporting Information

Table S1 Time from Ethical Approval to Publication for Phase I Versus Phase II–IV Trials (21 KB XLS)

## Abbreviations

REC: research ethics committee
